# Estimation of Dynamic Bivariate Correlation Using a Weighted Graph Algorithm

**DOI:** 10.3390/e22060617

**Published:** 2020-06-02

**Authors:** Majnu John, Yihren Wu, Manjari Narayan, Aparna John, Toshikazu Ikuta, Janina Ferbinteanu

**Affiliations:** 1 Center for Psychiatric Neuroscience, Feinstein Institute of Medical Research, Manhasset, NY 11030, USA; 2 Division of Psychiatry Research, The Zucker Hillside Hospital, Northwell Health System, Glen Oaks, NY 11004, USA; 3 Department of Mathematics, Hofstra University, Hempstead, NY 11549, USA; Yihren.Wu@hofstra.edu; 4 Department of Psychiatry and Behavioral Sciences, Stanford University, Paolo Alto, CA 94305, USA; manjarin@stanford.edu; 5 Department of Electrical and Computer Engineering, The University of Texas at San Antonio, San Antonio, TX 78249, USA; aparnajohnutsa@gmail.com; 6 Department of Communication Sciences and Disorders, School of Applied Sciences, University of Mississippi, Oxford, MS 38677, USA; tikuta@olemiss.edu; 7 Departments of Physiology and Pharmacology and of Neurology, State University of New York Downstate Medical Center, Brooklyn, NY 11203, USA

**Keywords:** dynamic bivariate correlation, dynamic correlation, fMRI, local field potential, sliding window, dynamic conditional correlation, functional connectivity

## Abstract

Dynamic correlation is the correlation between two time series across time. Two approaches that currently exist in neuroscience literature for dynamic correlation estimation are the sliding window method and dynamic conditional correlation. In this paper, we first show the limitations of these two methods especially in the presence of extreme values. We present an alternate approach for dynamic correlation estimation based on a weighted graph and show using simulations and real data analyses the advantages of the new approach over the existing ones. We also provide some theoretical justifications and present a framework for quantifying uncertainty and testing hypotheses.

## 1. Introduction

Dynamic bivariate correlation, or dynamic correlation for short, is correlation between a pair of time series, which itself changes over time. Assessing dynamic correlation is of importance in many areas within neuroscience. Dynamic correlation estimation is of interest in neuroimaging because many studies have identified dynamic changes in functional connectivity during the course of a functional magnetic resonance imaging (fMRI) experiment [1,2,3,4,5,6,7], especially during resting state. Estimating dynamic correlation is of interest in neurophysiology as well. For example, dynamic correlation between local field potential time series obtained from different brain regions could be used to explore how certain brain regions work in tandem during certain specific behaviors. When identifying such changes, it is of importance to make sure that the dynamic shifts in the correlations observed are not due to spurious fluctuations inherent to the estimation method used.

A commonly used method for dynamic correlation estimation is the sliding window (SW) method. Although easy to understand and implement, the main limitation of the SW method is uncertainty regarding the choice of the window size. Another well-established method for dynamic correlation estimation is dynamic conditional correlation (DCC) developed by Engle and Sheppard in the field of econometrics [8,9] and widely used in finance literature. A few years ago DCC was introduced as a useful tool for neuroimaging researchers by Lindquist and co-authors [10]. Although DCC has many desirable features compared to SW method, it has certain limitations.

Time series measurements occurring in neuroscience literature occasionally have a few extreme values, as seen, for example, in the local field potential time series data, which is one of the data sets analyzed in this paper. Such values can be ignored only if they are an anomaly that does not make sense with the underlying scientific framework. In this paper, among other things, we show that the performance of DCC is not robust in the presence of extreme values. Another limitation of DCC is related to its practical implementation using numerical algorithms. Optimization procedures in DCC parameter estimation involves inversion of matrices which could lead to numerical instability. Although this computational issue is compounded in the presence of extreme values, it could occasionally be an issue even when there are no extreme values present in the pair of time series under consideration.

To address these problems, we propose a novel, robust approach for estimating dynamic correlation estimation. Our method is based on converting time series into a weighted graph and hence we refer to it as weighted graph algorithm (WGA). We illustrate the utility of the new method via simulations and real data examples. We also present theoretical justifications and provide a framework for calculating confidence intervals and conducting hypothesis tests for dynamical correlation based on the WGA approach.

## 2. Methods

We consider three methods for estimating dynamic correlation in this paper. The first two methods—the SW and DCC—are currently existing methods. The third method based on a weighted graph algorithm (WGA) is a new approach to the dynamic-correlation-estimation problem. We illustrate the methods using two time series generated as in Lindquist et al’s [10] simulations study #1. Random data were generated for each time point using a mean-zero bivariate normal distribution, with covariance matrix set to

$$\left[\begin{array}{cc} \sqrt{2} & 0\\ 0 & \sqrt{3} \end{array}\right].$$

**Figure 1:**
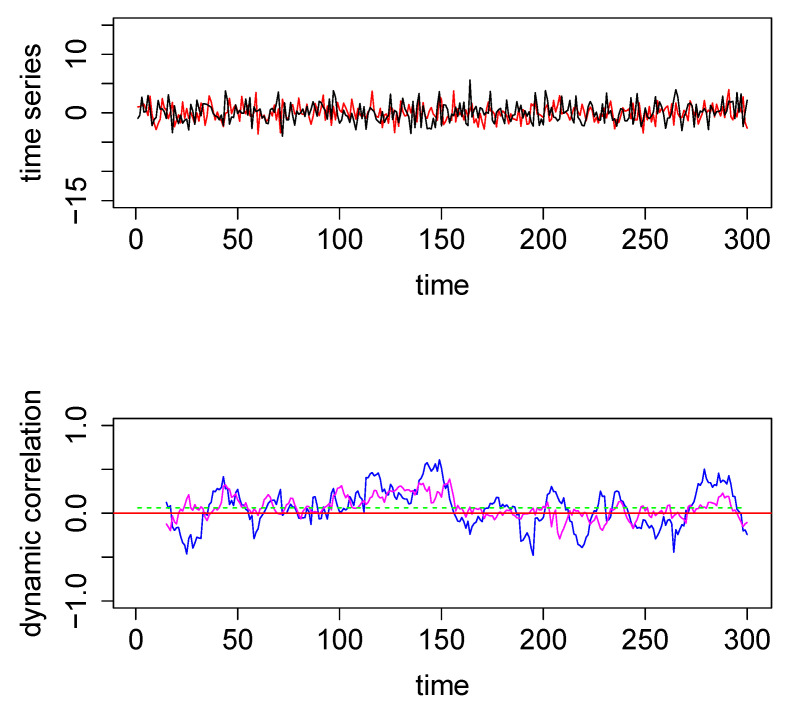
**Top panel**: The two time series used for the first illustrative simulated example; data were generated from a bivariate normal distribution. **Bottom Panel**—Dynamic correlations estimated for the above two time series using 3 different methods—method #1, sliding window (SW) (blue); method #2, dynamic conditional correlation (DCC) (green) (see also Figure A1); and method #3, weighted graph algorithm (WGA) (magenta). The red horizontal line in the center through zero represents the underlying true dynamic correlation for this example.

A bivariate time series generated using the above mechanism with T=300 time points is shown in the top panel in Figure 1 below. The dynamic correlation was *a priori* set to zero in this simulated example. That is, the underlying correlation equals zero at all time points in this simulated bivariate time series.

### 2.1. Method # 1—Sliding-Window (SW) Technique

SW technique [1,2,4] is easy to explain and implement. We choose a window-frame of size ws = 15 for the example considered in Figure 1. At the first iteration, we used the window-frame to frame the first (ws=) 15 time points of the bivariate time series, and find the correlation between the values of the two series within this window-frame. Then we repeat the process by sliding the window to the right, one unit at a time. Thus, in general, at iteration *i*, the right-end of the SW is placed at the ${\left(i+ws\right)}^{th}$ time point of both time series, and the correlation between the two time series within the window-frame was calculated. The series of correlations obtained as the right-end of the SW is moved from the $ws=15^{th}$ time-point all the way to $T=300^{th}$ time-point of the two time series form the blue curve plotted in the bottom-panel of Figure 1.

To make it clearer, the 1st correlation value is the correlation between two vectors each of length 15: the 1st and the 2nd vectors containing the values corresponding to time points between 1 and 15 (end points included) of the 1st and the 2nd time series, respectively. To obtain the second correlation, we move the window one unit to the right, so that for this second iteration the right-end of the window-frame is at time-point 16, and the left end is at time-point 2. Thus the 2nd correlation value will be the correlation between two vectors each of length 15: the 1st and the 2nd vectors containing the values corresponding to time points between 2 and 16 (end points included) of the 1st and the 2nd time series, respectively. We continue moving the SW one unit to the right at each iteration and calculating the corresponding correlation until the final iteration (that is, when the right-end of the SW is at $T=300^{th}$ time point). All the consecutive correlations thus obtained are plotted as the blue curve in the bottom-panel. The performance of this method depends on the size of the window: a larger ws provides smoother estimates of dynamic correlation, while smaller ws is more sensitive to variations in the underlying data. Lindquist et al. [10] discusses this issue in more detail, and also mentions variations of the above technique such as tapered sliding window [1].

### 2.2. Method # 2—Dynamic Conditional Correlation

Here we outline the key steps involved in the DCC method. A good source with a detailed exposition is [11]. Let us denote the two time series under consideration by $\left\{y_{1,t}\right\}$ and $\left\{y_{2,t}\right\}$. If the conditional correlation between $\left\{y_{1,t}\right\}$ and $\left\{y_{2,t}\right\}$ at time *t* is denoted by $\rho_{t}$, then the corresponding correlation matrix may be denoted by $R_{t}$:

$$R_{t}=\left[\begin{array}{cc} 1 & \rho_{t}\\ \rho_{t} & 1 \end{array}\right].$$

If the conditional variances at time *t* for the two series $\left\{y_{1,t}\right\}$ and $\left\{y_{2,t}\right\}$ are $\sigma_{1,t}^{2}$ and $\sigma_{2,t}^{2}$, respectively, then the corresponding conditional variance-covariance matrix

$$\Sigma_{t}=\left[\begin{array}{cc} \sigma_{1,t}^{2} & \rho_{t}\sigma_{1,t}\sigma_{2,t}\\ \rho_{t}\sigma_{1,t}\sigma_{2,t} & \sigma_{2,t}^{2} \end{array}\right],$$

which may also be written as

$$\Sigma_{t}=\left[\begin{array}{cc} \sigma_{1,t} & 0\\ 0 & \sigma_{2,t} \end{array}\right]\left[\begin{array}{cc} 1 & \rho_{t}\\ \rho_{t} & 1 \end{array}\right]\left[\begin{array}{cc} \sigma_{1,t} & 0\\ 0 & \sigma_{2,t} \end{array}\right]=D_{t}R_{t}D_{t},\;\mathrm{where}\;D_{t}=\left[\begin{array}{cc} \sigma_{1,t} & 0\\ 0 & \sigma_{2,t} \end{array}\right],$$

or equivalently $R_{t}=D_{t}^{-1}\Sigma_{t}D_{t}^{-1}$. If we denote

$$y_{t}=\left[\begin{array}{c} y_{1,t}\\ y_{2,t} \end{array}\right]=\left[\begin{array}{c} \mu_{1,t}\\ \mu_{2,t} \end{array}\right]+\left[\begin{array}{c} e_{1,t}\\ e_{2,t} \end{array}\right]=\mu_{t}+e_{t},\;\mathrm{where}\;E\left(y_{t}\right)=\mu_{t}\;\mathrm{and}\;E\left(e_{t}\right)=0,$$

and without loss of generality assume $\mu_{t}=0={\left[0,0\right]}^{\prime }$ (–otherwise, we can always center the time series data at the respective means to make this assumption valid) then $\Sigma_{t}=E\left(y_{t}y_{t}^{\prime }\right).$ Here the prime symbol ${}^{\prime }$ right next to a vector or a matrix denotes its transpose. Hence,

(1) $$E\left(\left[D_{t}^{-1}y_{t}\right]{\left[D_{t}^{-1}y_{t}\right]}^{\prime }\right)=D_{t}^{-1}E\left(y_{t}y_{t}^{\prime }\right)D_{t}^{-1}=D_{t}^{-1}\Sigma_{t}D_{t}^{-1}=R_{t}.$$

Thus, if we can somehow estimate $D_{t}$ (that is, estimate $\sigma_{1,t}$ and $\sigma_{2,t}$) as a first step, then we may determine $D_{t}^{-1}y_{t}=r_{t}$. Furthermore, the sample variance-covariance matrix of $r_{t}$ will be an estimate of $R_{t}$ based on Equation (1). Hence the first question is: ‘Is there a way to estimate $\sigma_{1,t}$ and $\sigma_{2,t}$?’ Extensive work in the econometrics field has shown that one of the best ways to address this question is using generalized autoregressive conditional heteroscedasticity (GARCH) modeling [12,13,14]. In econometrics literature the standard deviations $\sigma_{1,t}$ and $\sigma_{1,t}$ are known as ‘volatilities’ of the series $\left\{y_{1,t}\right\}$ and $\left\{y_{2,t}\right\}$ at time *t*, respectively, and $r_{t}$ is known as ‘volatility-adjusted data’. In the GARCH(1,1) setting $\sigma_{i,t}$ for each i=1,2 is estimated recursively as the weighted sum of the unconditional estimated variance at t−1 (i.e., $y_{i,t-1}^{2}$) and $\sigma_{i,t-1}^{2}$:

$$\sigma_{i,t}^{2}=\omega_{i}+\alpha_{i}y_{i,t}^{2}+\beta_{i}\sigma_{i,t-1}^{2},\;i=1,2.$$

Thus the six parameters to be estimated in this model are $\omega_{i},\alpha_{i},\beta_{i},\;i=1,2.$ As mentioned before, once the first step is completed we will have an estimate of $D_{t}$ with which we will be able to calculate $r_{t}=D_{t}^{-1}y_{t}$ (the latter calculation is known as “DE-GARCHING" in econometrics literature). In the next step we estimate the 2×2 variance-covariance matrix of $r_{t}$ using a recursive approach (-similar to the recursive approach in GARCH (1,1))

(2) $$Q_{t}=I+ar_{t-1}r_{t-1}^{\prime }+bQ_{t-1},$$

as a weighted sum of the contribution $r_{t-1}r_{t-1}^{\prime }$ at t−1, and the estimate $Q_{t-1}$ that we obtained by t−1. I is an intercept matrix which has ones in the diagonal. Ideally, we should be done by the end of this second step because based on the theory we know that $Q_{t}$ is an estimate of $R_{t}$. However, in practice $Q_{t}$ may deviate slightly from satisfying all the properties of a correlation matrix. Hence $Q_{t}$ is labelled as a quasi-correlation matrix. Note that the number of parameters to be estimated in Equation (2) are three: *a*, *b* and the one off-diagonal parameter in the symmetric matrix I. Although we deal with only two time series here, all the steps in the DCC algorithm can be easily extended to the case of *N* time series, N≥2. For larger *N*, the number of parameters in I, N(N−1)/2 can get very large. An alternate method (known as first-order correlation targeting) used in practice is to estimate of I using

$$\hat{I}=\left(1-a-b\right)\hat{S},\;\mathrm{where}\;\hat{S}=\frac{1}{T}\sum_{t=1}^{T}r_{t-1}r_{t-1}^{\prime }$$

is the sample unconditional correlation matrix of the standardized residuals. If we use $\hat{I}$ instead of I, then in Equation (2) we will have 2 parameters rather than 2+[N(N−1)/2] parameters. Although first-order correlation targeting helps computationally, it may induce a small downward bias in *a* for larger *N*. There are methods to correct this bias, but for N=2 the bias is negligible and hence in this paper we will apply correlation targeting in the second step without bias correction. For general *a* and *b*, Equation (2) models a mean-reverting process. Typically, for most pairs of time series occurring in finance, changes in correlations appear to be temporary and mean-reverting. However, this need not be always the case for time series pairs in neuroimaging or neuroscience literature. To model such a scenario, an alternate to Equation (2) could be used:

$$Q_{t}=\lambda r_{t-1}r_{t-1}^{\prime }+\left(1-\lambda \right)Q_{t-1},$$

a process which has no tendency to have correlations revert to a constant value and should be particularly useful in modeling correlation that jumps one or more times.

The matrix $Q_{t}$ estimated in the second step may not yield a proper correlation matrix in practice (e.g., certain elements could be outside (−1,1)). Thus, in the final and third step of DCC, $Q_{t}$ is rescaled to get a proper estimate of $R_{t}$:

$$R_{t}=\mathrm{diag}{\left\{Q_{t}\right\}}^{-1/2}Q_{t}\mathrm{diag}{\left\{Q_{t}\right\}}^{-1/2}.$$

The most commonly used version of DCC, outlined above, may be summarized as follows:

1st step:
  - Estimate $\sigma_{i,t}^{2}$ using GARCH(1,1) $\sigma_{i,t}^{2}=\omega_{i}+\alpha_{i}y_{i,t}^{2}+\beta_{i}\sigma_{i,t-1}^{2}$
  - DE-GARCH data: $r_{t}=D_{t}^{-1}y_{t},\;\mathrm{where}\;D_{t}=\mathrm{diag}\left\{\sigma_{1,t},\sigma_{2,t}\right\}.$
2nd step:
  - Estimate quasi-correlation matrix $Q_{t}=\left(1-a-b\right)\hat{S}+ar_{t-1}r_{t-1}^{\prime }+bQ_{t-1}$, where $\hat{S}=\frac{1}{T}\sum_{i=1}^{T}r_{t-1}r_{t-1}^{\prime }$
3rd step:
  - Rescale to get a proper correlation matrix: $R_{t}=\mathrm{diag}{\left\{Q_{t}\right\}}^{-1/2}Q_{t}\;\mathrm{diag}{\left\{Q_{t}\right\}}^{-1/2}.$

Estimation of the parameters in practice is done using a 2-step process under the quasi-maximum-likelihood framework. The log-likelihood *L* can be split as a sum $L=L_{1}+L_{2}$, where

$$L_{1}=-\frac{1}{2}\sum_{t=1}^{T}\left[n\log\left(2\pi \right)+2\log|D_{t}|+y_{t}^{\prime }D_{t}^{2}y_{t}\right]\;\mathrm{and}\;\;L_{2}=-\frac{1}{2}\sum_{t=1}^{T}\left[\log|R_{t}|+r_{t}^{\prime }R_{t}^{-1}r_{t}-r_{t}^{\prime }r_{t}\right].$$

$L_{1}$ consists of only variance parameters and $L_{2}$ consists of only correlation parameters, thereby facilitating a 2-step process. In the first step, the variance part of *L* (i.e., $L_{1}$) is maximized to obtain estimates of the corresponding parameters and based on these estimates standardized residuals $r_{t}$’s are obtained. In the second step the standard residuals from the first step is plugged-in into $L_{2}$ and then $L_{2}$ is then maximized with respect to the correlation parameters. Note that the second step involves inversion of the full matrix $R_{t}$ for each *t*; this could lead to non-convergence of the numerical optimization involved in estimation. Such numerical issues are aggravated when *N* is large, but they are less of a problem for normal data points when *N* = 2. However, even when *N* = 2, in the presence of extreme values in the data, the corresponding matrices may get close to singular which could lead to numerical instability and inaccurate results. All DCC analysis for this paper was conducted in Matlab using the DCC toolbox (https://github.com/canlab/Lindquist_Dynamic_Correlation).

### 2.3. Method # 3—Weighted Graph Algorithm (WGA)

The new approach proposed in this paper is best explained using a fully connected weighted graph associated with each time series. For a given time series with values $x\left(t_{i}\right)=x_{t_{i}},i=1,\ldots T,$ we consider a graph with *T* nodes, where the ith node $n_{i}$ corresponds to the ith time point $t_{i}$. Any pair of nodes ($n_{a},n_{b}$) (corresponding to the pair of time points ($t_{a},t_{b}$)) is connected by an edge with a weight $w_{ab}$ associated with it. We define the weight $w_{ab}$ to be

$$w_{ab}=\arctan\frac{x\left(t_{b}\right)-x\left(t_{a}\right)}{t_{b}-t_{a}}.$$

Our graph construction is inspired by, yet different from, the Weighted Visibility Graphs (WVGs) presented in Supriya et al. [15]. WVGs are weighted versions of visibility graphs introduced by Lacasa et al. [16]. Visibility graph algorithm, which converts a time series into a graph based on a visibility criterion, has found wide applications in physics and many other fields. (See Appendix B for more details about the visibility criterion defined by Lacasa et al). Methods based on visibility graphs became popular after the original concept of mapping time series to a complex network was introduced in Zhang and Small [17]. The main difference between the above-mentioned constructions from existing literature and ours is that we do not use a visibility criterion, and hence, in particular, our graph is fully connected.

Our Weighted Graph Algorithm (WGA) for dynamic correlation estimation is also based on a sliding window. As done for method 1, we pick a window of size 15 for illustration. Associated with each time point $t_{i}$ (that is, with each node $n_{i}$ in the WGA graph) is a weight-vector of length *T* consisting of the weights of the edges from all the other nodes of the graph to $n_{i}$. We named this weight vector as $w_{i}$. Note that we considered the weight between $n_{i}$ to itself as zero. That is, ith element of $w_{i}$ is zero, for all *i*. For each time series, we place the right-end of a sliding window (of size 15) at time point *i*, considered all the weight vectors

$$\left\{w_{i-15+1},w_{i-15+2},\ldots ,w_{i}\right\},$$

and obtained a new vector $W_{\left(i:\;\mathrm{median}\right)}$ by taking the median element-wise. That is kth element of $W_{\left(i:\;\mathrm{median}\right)}$ is the median of the kth elements of $\left\{w_{i-15+1},w_{i-15+2},\ldots ,w_{i}\right\}.$ The steps so far are illustrated in the schematic diagram presented in Figure A7 in the Appendix B.

If we use super-scripts to denote the vectors corresponding to the two time series, then the above steps may be summarized using the following formula:

$$W_{\left(i:\;\mathrm{median}\right)}^{\left(j\right)}\left[k\right]=\mathrm{median}\left\{w_{i-15+1}^{\left(j\right)}\left[k\right],w_{i-15+2}^{\left(j\right)}\left[k\right],\ldots ,w_{i}^{\left(j\right)}\left[k\right]\right\},$$

where k(=1,…,T) denotes the kth element of each vector and j(=1,2) denote the jth time series. The dynamic correlation estimate at the ith time point (where *i* ranges from 15 to *T*) in our approach is the correlation between the vectors $W_{\left(i:\;\mathrm{median}\right)}^{\left(1\right)}$ and $W_{\left(i:\;\mathrm{median}\right)}^{\left(2\right)}$. A heuristic justification and further theoretical considerations are provided in the Appendix C.

*Illustration of the Methods*

**Figure A1:**
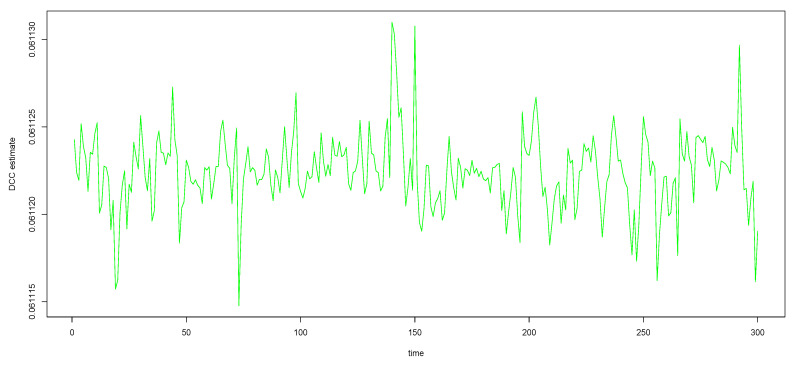
The green curves (DCC estimates) in the bottom panel of Figure 1, with *y*-axis zoomed in, to see that the curves are not really straight lines.

To illustrate graphically how the three methods perform, we computed the three dynamical correlations and plotted them together in the bottom panel of Figure 1. The underlying dynamic correlation, which is equal to zero at all time points, is plotted as the red line. The correlations obtained through the SW, WGA and DCC methods are plotted as blue, magenta and green curves in bottom panel in Figure 1. The dynamic correlation estimates based on DCC (green curve) may appear to be a straight line; but this is not in fact the case (see Figure A1 in the Appendix A where we used a different scale for the y-axis). SW method (blue curve) results in the worst estimate, DCC method (green curve) results in the best estimate, and the WGA method (magenta curve) performs somewhere in between. This qualitative estimation is confirmed quantitatively by the following summary statistics: the maximum absolute value for the blue, magenta and green curves are, respectively, 0.61, 0.39, and 0.06.

**Figure 2:**
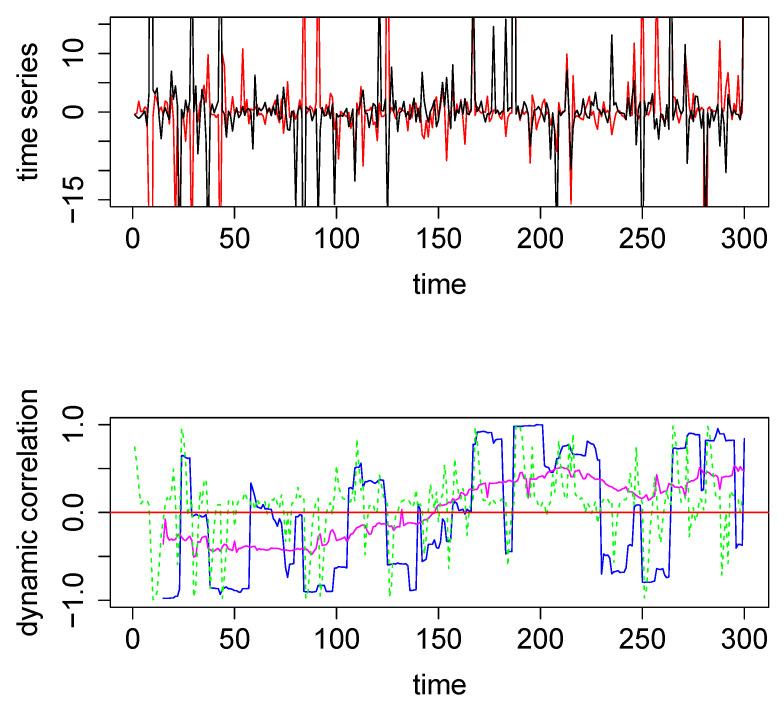
**Top panel**: The two time series used for the second illustrative simulated example; data were generated from a bivariate Cauchy distribution. **Bottom Panel**: Dynamic correlations estimated for the above two time series using various methods. Method #1, SW (blue), method #2, DCC (green), and method #3, based on WGA (magenta). The red horizontal line in the center through zero represents the underlying true dynamic correlation for this example.

**Figure A2:**
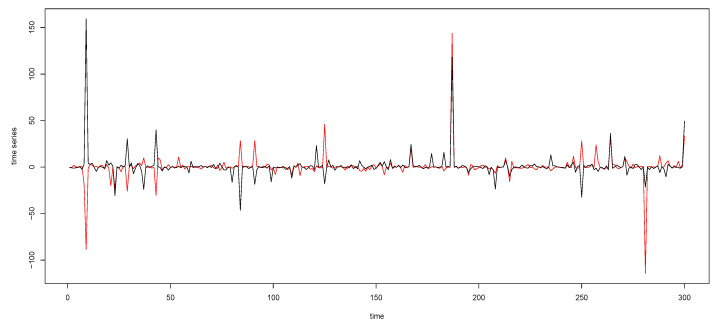
The time series plot in the top panel in Figure 2 with the *y*-axis margins expanded.

In the above example, DCC performs better than both the SW technique and the new method. However, the data in this example are a pair of nicely behaved time series with no outliers (since they were generated from a bivariate normal density). However, in the presence of outliers DCC does not perform as well. We generated two time series from a bivariate Cauchy density (by using the R function *rcauchy2d* from the package *fMultivar*) [18]. The correlation parameter for the bivariate Cauchy density (ρ) was set to zero, so that the underlying dynamic correlation for this example was also zero for all time points. The two time series thus generated are plotted in the top panel in Figure 2. Because Cauchy density has very heavy tails, the generated data contain several extreme values. We applied the same three analytical methods to this data set and the results are plotted in the bottom panel in Figure 2, with the same color coding as in Figure 1. For this example, the new method easily outperforms the other two methods, as seen visually. The maximum absolute values for the blue, green, and magenta curves are, respectively, 0.9986, 0.9989 and 0.5298, respectively. Thus the new method was more robust when extreme values were present in the data. As a minor note, the y-axes for the top panels had the same scale for Figure 1 and Figure 2, which cut off the plot for some extreme values in Figure 2. In order to see the actual extreme values, we plotted the same pair time series with extended y-margins in Appendix A Figure A2.

### 2.4. Simulation Design

To assess the performance of the three methods under various scenarios, we ran extensive simulations using scenarios aimed to closely match the work of Lindquist et al. [10]. We therefore generated data by using bivariate normal distribution, as in Lindquist et al. [10], but also bivariate Cauchy distribution, to obtain data sets with extreme values. In all the simulations involving bivariate Cauchy, values exceeding 50 were reset to 50, and values below –50 were reset to –50. The means for all pairs of time series in the simulation study was set to (0,0) across all time points and the covariance matrix at time point *t* was

(3) $$\left[\begin{array}{cc} 2 & \sqrt{6}p\left(t\right)\\ \sqrt{6}p\left(t\right) & 3 \end{array}\right].$$

By considering three different forms for p(t) we obtained three different simulations scenarios, as in Lindquist et al. [10]. For simulations involving the sliding window technique (method #1) and the WGA (method #3), the window size was set to 15.

*Simulations Design D1:* In this case, we set p(t)=0 for all *t* as in the illustrative examples above. We considered four time series of different lengths (T=150,300,600 and 1000) for both bivariate normal and bivariate Cauchy distributions. The metrics used to assess the performance of the methods for this scenario were the mean and maximum absolute value of correlations across all time points.

*Simulations Design D2:* The scenarios in D2 are designed to assess the performance of the methods when the underlying correlation varies slowly over time. We set $p\left(t\right)=\sin\left(t/\Delta \right)$, $\Delta =1024/2^{k}$, with k=3,4. We obtained two different (sub-)scenarios corresponding to k=3 and 4, which we refer to as D2a and D2b. We set T=600 for both of these sub-scenarios. The metric used to assess the performance was mean-squared-error (MSE) as in Lindquist et. al [10].

*Simulations Design D3:* The scenarios in D3 are designed to assess the performance of the methods when there is a rapid change in the underlying correlation; here, the rapid change occurs near time point 250. We set p(t) equal to a Gaussian kernel with mean 250 and standard deviation 15*k*, where *k* = 3 and 4. The two *k*’s resulted in two different (sub-)scenarios, D3a and D3b; T=600 for both cases. MSE was used to compare the performance of the methods.

### 2.5. Real Data Analysis

To illustrate the advantages and disadvantages of the methods considered in this paper, we applied our analysis to two different empirical data sets. The first data set used for illustration was fMRI data collected from one healthy adult subject while the subject performed a color-word Stroop task [19]. The data were part of a sample collected for a study of the underlying mechanisms based on distributed network of brain regions involved in congruency sequencing effect in cue-conflict paradigms [20]. Time series from six different brain regions were used in our analysis, and dynamic correlations between each pair of regions were obtained. The second data set used for illustration consisted of local field potential (LFP) time series [21,22] obtained from four different electrodes implanted in the brain of a rat. The recording occurred while the rat was awake and placed on a small platform in the lab. Three of the recordings were from the CA1 field of the hippocampus, and one recording from the medial dorsal striatum. Dynamic correlations of data from each pair of recordings were computed. To make the results of our analysis accessible, we did not apply any pre-processing to the recorded LFPs except downsampling from 2 kHz to 1 kHz. This procedure presented the movement artifacts which are prominent in the displayed signals. Although the behavior of the dynamic correlations of pairwise time series was of intrinsic interest for the background scientific research question related to both fMRI and LFP data sets, the main purpose in the current analysis was to illustrate various aspects/issues associated with the estimation methods considered in this paper. Specifically, the Stroop task fMRI data set illustrated the convergence issues related to DCC and LFP data illustrated the performance of the methods in the presence of extreme values. Details about data extraction for fMRI data set and recording for the LFP data are presented below. Codes used for all analysis and simulations are available at https://github.com/janinaf/WGA-DC.

*fMRI data*. Sample fMRI data from one healthy human adult subject were downloaded from OpenfMRI.org. We used Stroop task fMRI data (https://openfmri.org/dataset/ds000164/) [20], since activation patterns for the Stroop task is relatively well known. Preliminary data processing followed previous publication [23]. Using FMRIB Software Library (FSL) as well as Analysis of Functional NeuroImages (AFNI), anatomical volume was skull stripped, segmented (gray matter, white matter and CSF), and registered to the MNI 2mm standard brain. Processing of fMRI echo planar image (EPI) volume included the following. The first four EPI volumes were removed; transient signal spikes were removed; head motion was corrected; the volumes were smoothed with a 6mm FWHM Gaussian kernel; the volumes were resampled, spatially transformed and aligned to the MNI 2mm standard brain space; volumes with excess motion are scrubbed. In order to extract the time series for six regions chosen *a priori*, the frontal medial cortex, subcallosal frontal region, insular cortex, Heschl’s gyrus, and amygdala were defined using the Harvard-Oxford atlas and the Caudate Head was defined using WFU PickAtlas. The EPI time series was extracted within each of these six regions.

*LFP data*. The local field potential (LFP) data were recorded from an awake rat placed on a small platform. Previous to the recording, the animal had been implanted with a 16 tetrode hyperdrive assembly intended for recording in the CA1 field of the hippocampus, combined with two cannula/electrode systems (PlasticsOne, Inc) aimed bilaterally at the medial dorsal striatum. The tetrodes were gradually lowered in the CA1 layer across 8 to 10 days after recovery from surgery, and their tip was positioned based on the configurations of sharp waves and ripples. The electrodes aimed at the dorsal striatum were implanted directly in the position from which the recording was obtained (AP: + 2.5 mm, ML: +/–2.4 mm; DV: –5.4 mm; tilted 22 degrees from the vertical in the sagittal plane). The single wire electrodes located in the striatum and the 16 tetrodes were connected through a PCB to a group of four headstages with a total of 64 unity gain channels and 2 color LEDs for position tracking. The LFP signals were sampled at 2K Hz, amplified (1000 x), band-pass filtered (1–1000 Hz), digitized (30K Hz), and stored together with LED positions on hard disk (Cheetah Data Acquisition System, Neuralynx, Inc.). Subsequently the signal was downsampled to 1K Hz, in which form it was used for the current analysis.

## 3. Results

### 3.1. Simulations Results

*Simulations Design D1:* Results from simulations with design D1 are presented in Table 1 and Table 2. Table 1 presents the results when the pair of time series was generated from a bivariate normal distribution and Table 2 presents the results related to bivariate Cauchy. In the bivariate normal case, across all T values, DCC performed best, SW performed worst, and WGA performance was in between. In the bivariate normal case, DCC’s performance improved with larger T as measured both by bias and variance, while that of SW and WGA methods remained roughly the same for all T. In the bivariate Cauchy case, the SW method performed consistently worst across all T, while for the other two methods performance improved with larger T. At small T values, the WGA method performed better than the DCC method. As T got larger, the DCC performance overtook that of WGA, and at T = 1000, DCC performed better than WGA based on the metrics used in Table. Even when MSE was used as the metric for comparison, DCC performed better than WGA when T = 1000: [$\left(0.203^{2}\right)+\left(0.036^{2}\right)]\;=\;0.043$ for WGA versus [$\left(0.148^{2}\right)+\left(0.081^{2}\right)]\;=\;0.028$ for DCC. Thus, in the presence of extreme values, DCC is robust as long as the number of time points is large enough, for this particular simulation design; when the number of time points is small, WGA-based evaluation is preferable to DCC.

**Figure 3:**
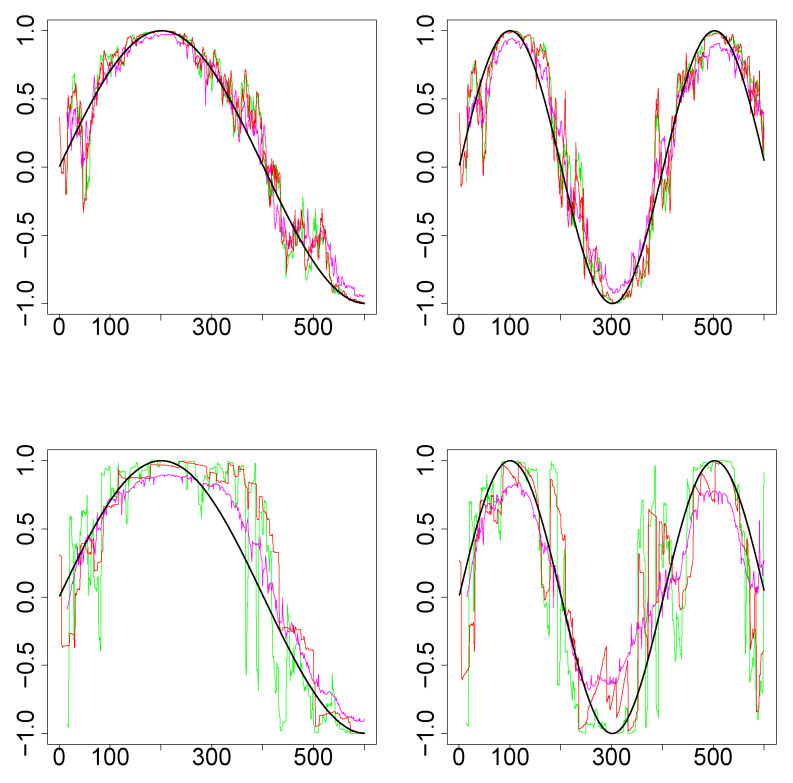
Results for single iteration from simulations design D2. (**Top row**): Underlying pair of time series from bivariate normal. (**Bottom row**): Underlying pair of time series from bivariate Cauchy. (**Left column**): Design 2a (that is, k=3). (**Right column**): Design 2b (k=4). The underlying true dynamic correlations are plotted as the black curve. Green (SW), magenta (WGA) and red (DCC) represents the estimates of dynamic correlation.

**Figure 4:**
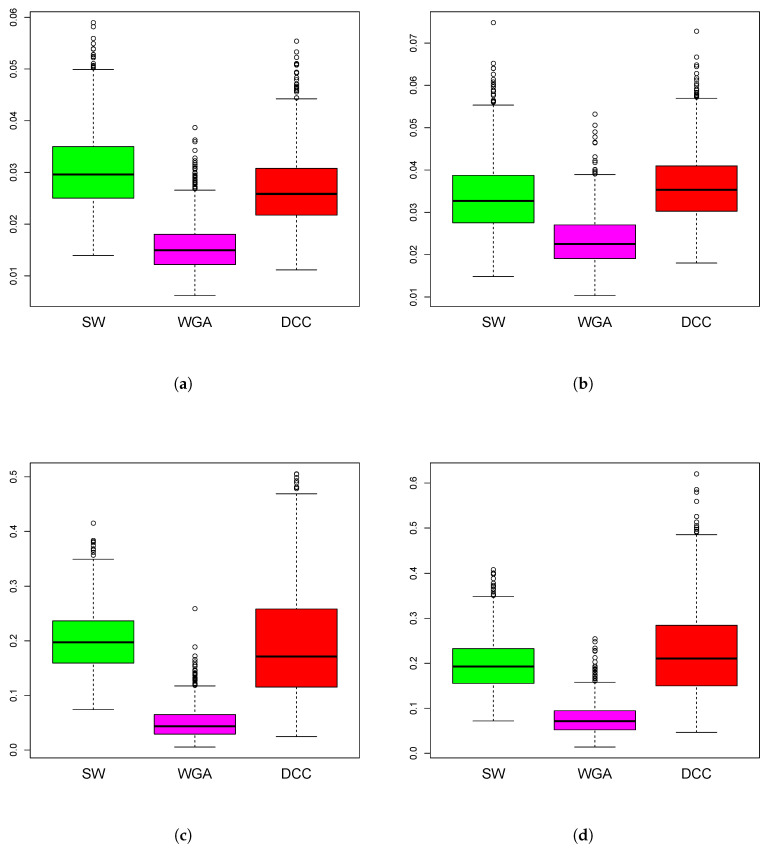
Boxplots of mean squared error of the estimation methods based on 1000 iterations from simulations design D2. The panels correspond to bivariate Normal (**a**,**b**), bivariate Cauchy (**c**,**d**), design D2a (**a**,**c**) and design D2b (**b**,**d**), as in Figure 3.

*Simulations Design D2:* In Figure 3, the black curves in the left panels are the underlying true dynamic correlations corresponding to simulations design D2a, the black curves in the right panels correspond to that of the simulations design D2b. The estimates of the dynamic correlations obtained via the three methods presented in this paper, for a single simulation iteration are plotted as colored curves. The top panels correspond to the bivariate normal scenario and the bottom panels correspond to the bivariate Cauchy scenario. Results from 1000 simulations for each scenario are plotted in Figure 4. In this design, the WGA-based method is far superior to the other two methods, in both bivariate normal and bivariate Cauchy cases.

**Figure 5:**
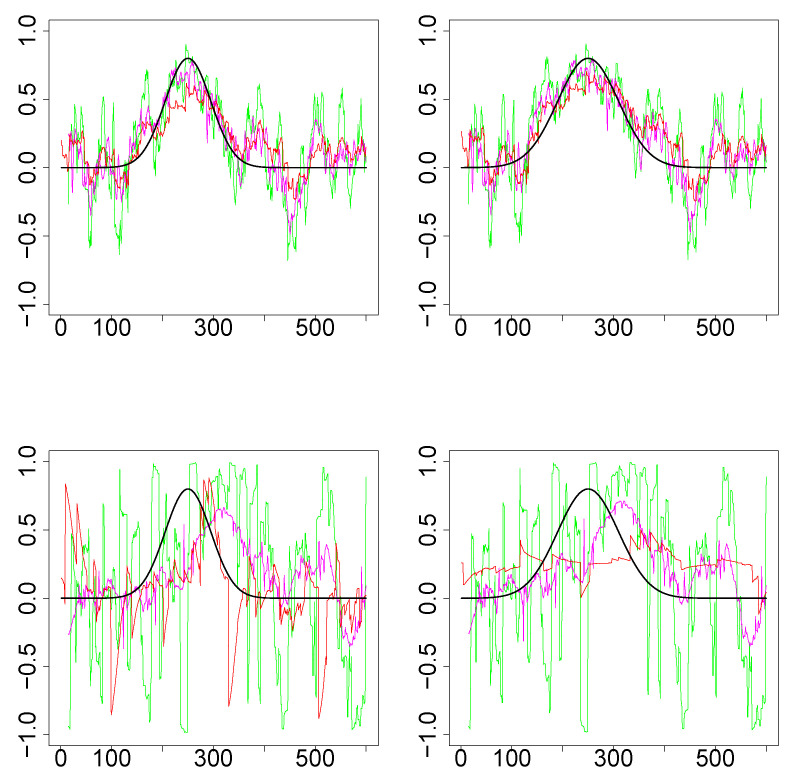
Results for single iteration from simulations design D2. (**Top row**): Underlying pair of time series from bivariate Normal. (**Bottom row**): Underlying pair of time series from bivariate Cauchy. (**Left column**): Design 3a (that is, k=3). (**Right column**): Design 3b (k=4). The underlying true dynamic correlations are plotted as the black curve. Green (SW), magenta (WGA) and red (DCC) represents the estimates of dynamic correlation.

**Figure 6:**
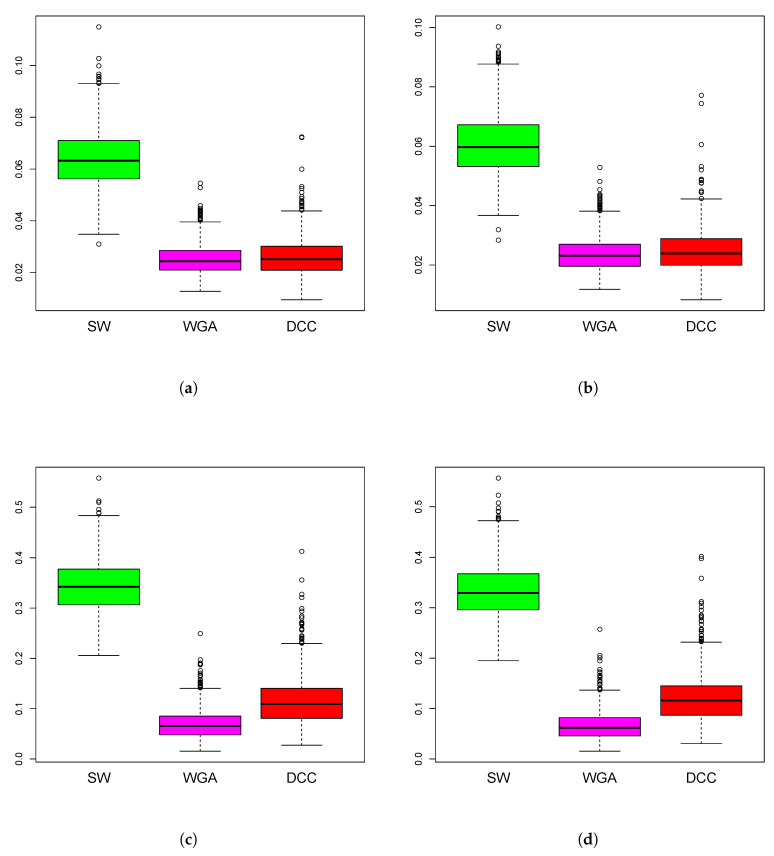
Boxplots of mean squared error of the estimation methods based on 1000 iterations from simulations design D3. The panels correspond to bivariate Normal (**a**,**b**), bivariate Cauchy (**c**,**d**), design D3a (**a**,**c**) and design D3b (**b**,**d**), as in Figure 5.

*Simulations Design D3: *Figure 5 plots the underlying true dynamic correlations and the estimates based on the three methods, for a single iteration corresponding to the simulation design D3. The results based on 1000 iterations are plotted in Figure 6. In all four scenarios for the design (D3a and D3b, bivariate normal and Cauchy), the performance of SW is substantially worse compared to the other two methods. DCC’s performance is comparable to that of WGA method in the bivariate normal scenario, although the WGA method has a slight edge over DCC. In the bivariate Cauchy case (bottom panels) the improvement in performance for the WGA-based method is more substantial. Thus, the results of the simulations indicate that when the underlying dynamic correlations are not constant across time, WGA performed better than DCC in many of the scenarios, and never worse than DCC in any of the scenarios considered.

### 3.2. Stroop Task Data Results

**Figure 7:**
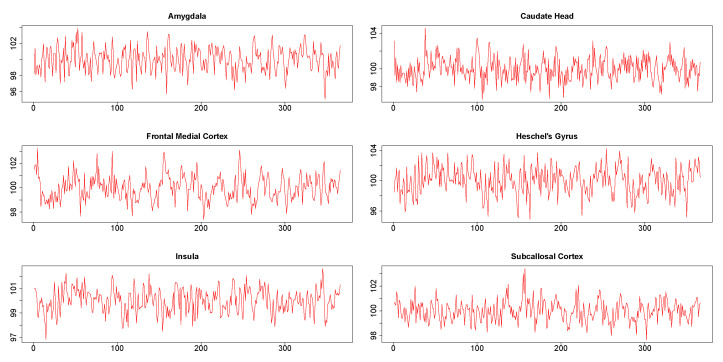
Time series from six regions in Stroop task functional magnetic resonance (fMRI) data.

The six times series obtained from six different brain regions of a single subject are shown in Figure 7. It may be noted in particular that the plots did not show the presence of any extreme values. The results from this data analysis are useful in illustrating certain numerical issues that arises during practical implementation of DCC.

**Figure 8:**
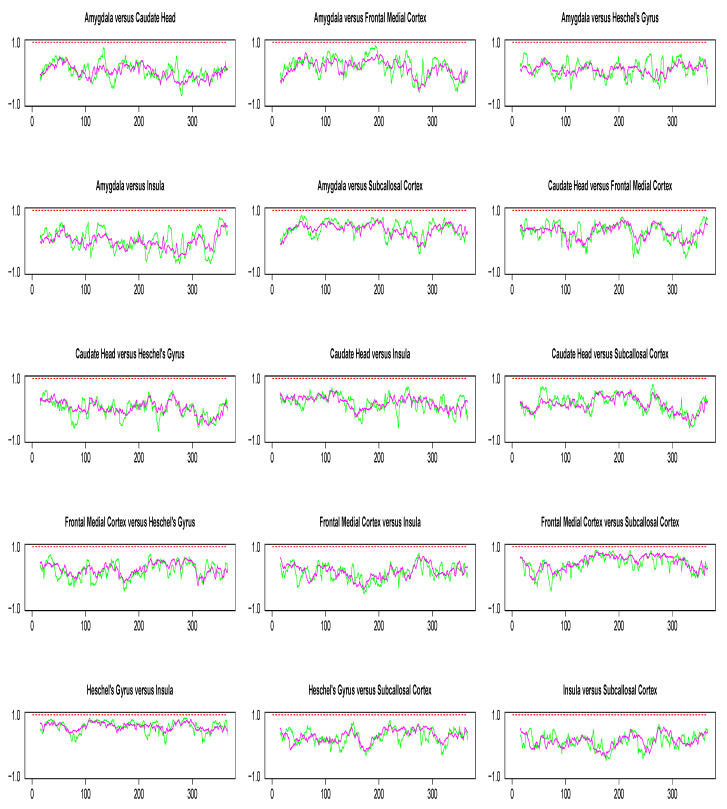
Pairwise dynamic correlation estimates for the Stroop task fMRI data. Green (SW), magenta (WGA), red (DCC).

Pairwise dynamic correlation plots are shown in Figure 8. In the absence of extreme values, the conclusions from our simulation study suggest DCC method to be the best candidate for dynamic correlation. However, as seen from Figure 8, the DCC estimate obtained in each pairwise analysis is consistently a flat line with mean value near 1. Since there is no biological explanation for such a result, our best guess is that it is due to the limitations of numerical recipes required for the implementation of optimization algorithms involved in DCC estimation. Such numerical issues did not occur in the implementation of the WGA method.

The data analysis presented in this subsection was mainly to illustrate the performance of the different estimation methods for dynamic correlation. Since the data presented originated from only one subject, not much can be read, interpreted or generalized from the results. Nevertheless, the highest correlation occurred between Heschel’s gyrus and insula (see Table 3 for the mean correlation across all time points for all pairwise analyses and Table A1 in the Appendix A for the corresponding maximum values of the correlations). Heschel’s gyrus contains the primary auditory cortex (PAC), the first cortical structure to process incoming auditory information [24]. In turn, PAC is in close proximity to the posterior insular cortex [24], to which it is also directly connected [25]. If we use the estimate of dynamic correlation as a proxy measure of functional connectivity, the data would imply that the Heschel’s gyrus and insula are functionally interconnected. This conclusion has clinical relevance because reduction in the functional connectivity between PAC and insula has been linked to prosody dysfunction in patients with schizophrenia [26].

*LFP Data Analysis Results*

**Figure 9:**
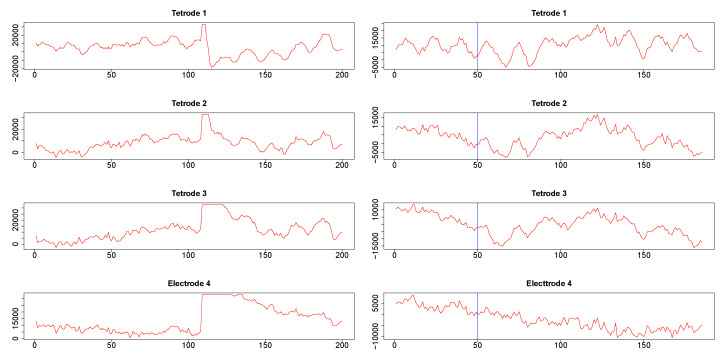
Segments of local field potential (LFP) time series from four electrodes implanted in the CA1 field of the hippocampus (tetrodes 1-3) and medial dorsal striatum (electrode 4) in the brain of the same rat. **Left column**: Segments between time points 200 and 400 from the respective time series plotted in appendix Figure A3. **Right column**: segments between time points 415 and 600 of time series seen in Figure A3. 50*^th^* time point within these segments is marked by a blue vertical line.

**Figure A3:**
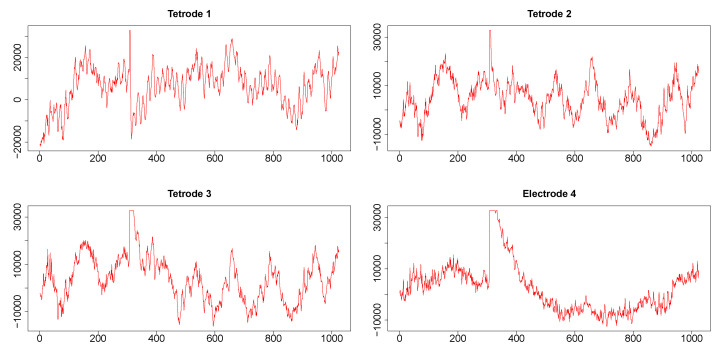
LFP time series related to two consecutive time stamps (1024 data points sampled at 1 KHz, approximately 1 sec) from three tetrodes and one electrode. From each of these time series, segments between time points 200 and 400, and between 415 and 600 were cut out and plotted in left and right panels, respectively, of Figure 9. These segments were analyzed for illustrating the dynamic correlation estimation methods.

Time series corresponding to 1 s (1024 data points) from the three tetrodes and one single wire electrode are plotted in appendix Figure A3. Tetrodes 1, 2 and 3 were placed in the CA1 field of the hippocampus, while electrode 4 was located in the medial dorsal striatum. All hippocampal tetrodes recorded a theta oscillation (7–14 Hz) typical for this brain area. Signals on tetrodes 2 and 3 were most similar, and included, along with theta, oscillations of much higher frequency; these oscillations were missing in the signal recorded on tetrode 1. The signal on electrode 4, recorded in the medial dorsal striatum, lacked the theta rhythm and was visibly different from the other three LFP’s. However, in all four recordings a large jump corresponding to a movement artifact occurred approximately near the 300th time point. The configurations of the four signals were used to evaluate the performance of SW, DCC, and WGA methods discussed above. To estimate the dynamic correlations between the signals in the presence of extreme values, we cut out segments of data between time points 200 and 400 for each of the four time series (left panels of Figure 9). We expected to obtain high correlations between all segments around the 300th data point, where the movement artifact registered in all four recordings. Outside of this point, we expected high correlation between LFPs 2 and 3, somewhat lower correlation between LFP 1 and either LFP 2 or LFP 3, and low correlations between LFP 4 and the signals on any of the three tetrodes. Furthermore, because of the presence of extreme values, we also expected that WGA would generate the best estimate.

Second, we also cut segments between time points 415 and 600 (plotted in the right panels in Figure 9). Visual inspection suggested a change in signal configuration around the 50th point of these fragments which should be reflected in changes in the dynamic correlations among LFPs 1-3 (left vs. the right of the 50th time point in Figure 9, right panels). At the same time, we expected little correlation between any of the LFPs 1-3 on one hand and LFP 4 on the other hand.

**Figure 10:**
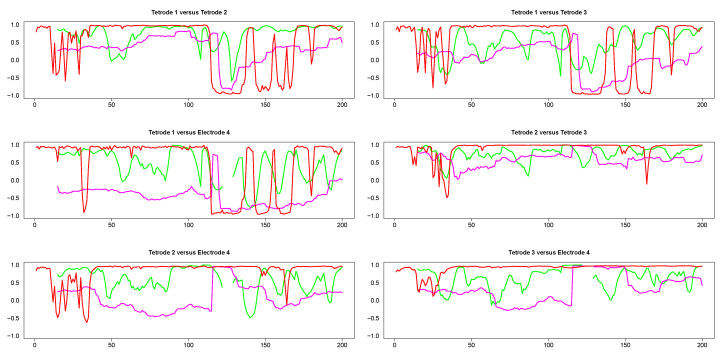
Pairwise dynamic correlations for time series plotted in the left column panels in Figure 9. Green (SW), red (DCC) and magenta (WGA).

The results from pairwise analyses of dynamic correlations for the segments in the left side of Figure 9 are plotted in Figure 10. In all these plots, the estimates generated by the SW and DCC methods seem to be fluctuating very rapidly compared to WGA, suggesting that the presence of extreme values affected SW and DCC much more than WGA. It is also interesting to note that the SW method did not always result in a correlation value (see discontinuities in the green curve in Figure 10) because in some portions the original signals consisted of series of equal numbers with zero standard deviation. Thus, the WGA method generated the most reasonable estimates for this set of time series.

**Figure A4:**
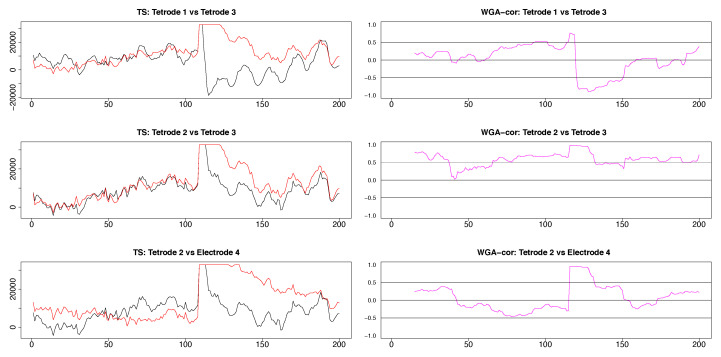
Three plots selected from the pairwise plots in Figure 10. LFP pairs are shown in the left column, the resulting dynamic correlations computed by using the WGA method are shown in the right column. In all cases, the maximum correlation occurs at the time point corresponding to the stimulus artifact. Overall, the highest correlations resulted for the tetrodes 2 and 3 LFPs which are the most similar, while the dynamic correlation between the very different LFPs of tetrode 2 and electrode 4 were dominated by low values.

Visual comparison of LFPs 1 and 3 (first and third left panels in Figure 9 top left panel in Figure A4) indicated that although both signals were dominated by the theta rhythm and showed a movement artifact, they were overall dissimilar, particularly at the later time points. The WGA estimates (top right in Figure 10, top right in Figure A4) reflected accurately these variations, showing low correlation at the beginning of the signals raising to a maximum correlation at the point of the extreme values corresponding to the movement artifact, dipping to negative values in the immediate aftermath of the peaks, and returning to positive values at the very end. Similarly, visual inspection of the tetrodes 2 and 3 signals (middle two panels in the left side of Figure 9; middle left panel in Figure A4), suggested consistent high correlations across all time points within the segments. The results of the WGA analysis showed that this was indeed the case (magenta curve in the middle right panel of Figure 10; middle right panel of Figure A4). There was no significant dynamic shift for the magenta curve and its values were above 0.5 for a significant portion of the segment. In contrast, the other estimates fluctuated rapidly (green and red curves, middle right panel in Figure 10) which neither makes sense biologically, nor verifies our understanding based on visual inspection of the original pair of LFPs. Finally, the WGA estimate indicated that the dynamic correlation between any of the hippocampal signals (LFPs 1–3) and the striatal signal (LFP4) was consistently low except in the section of extreme values, where, not surprisingly, the values are high (middle left and bottom both panels in Figure 10, bottom right in Figure A4). This assessment corresponded with our expectation that the LFPs originating in the two different brain areas would be uncorrelated.

**Figure 11:**
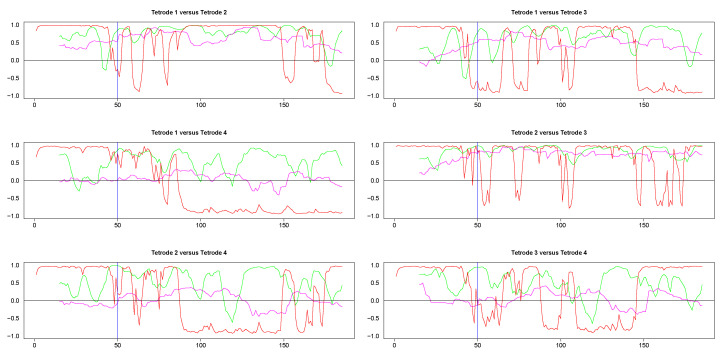
Pairwise dynamic correlations for time series plotted in the right column panels in Figure 9. Blue (SW), magenta (WGA), green (DCC). 50*^th^* time point within these segments is marked by a light blue vertical line.

**Figure A5:**
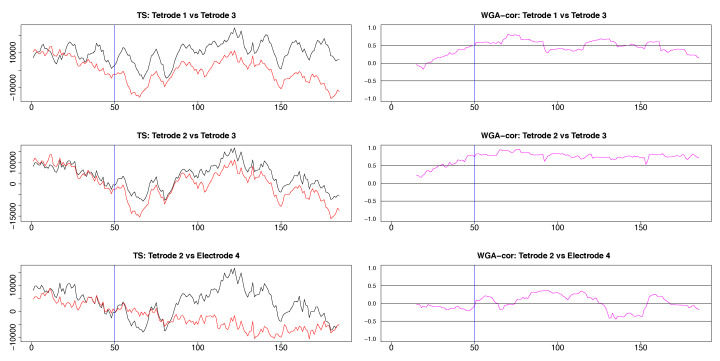
Three plots selected from the pairwise plots in Figure 11. LFP pairs are shown in the left column, the resulting dynamic correlations computed by using the WGA method are shown in the right column. The hippocampal LFPs become more similar after the 50th time point and the dynamic correlations in the top two panels reflect adequately this change. The LFP’s recorded in hippocampus and medial dorsal striatum are very different, a characteristic reflected in the low values of the corresponding dynamic correlation.

**Figure A7:**
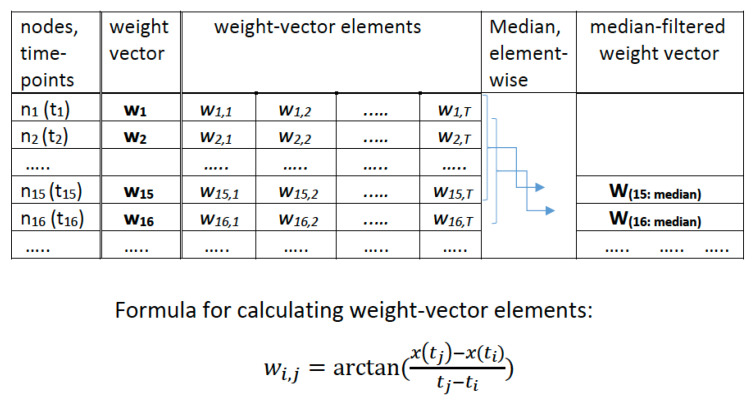
Schematic diagram illustrating the median-filtered weight vectors used in the algorithm presented in this paper.

The results from pairwise analyses of dynamic correlations for the segments in the right side of Figure 9 are plotted in Figure 11 and Figure A5. As in the previous case, the estimates based on SW and DCC fluctuated rapidly. In contrast, WGA analysis reflected correctly the switch in dynamic correlation before and after the 50th time point and the higher similarity between the signals on tetrodes 2 and 3 vs. signal on tetrode 1 (top left panel and top two right panels in Figure 11, top two rows in Figure A5). Except for the segment before the 50th point, the WGA estimate of the dynamic correlation between tetrodes 2 and 3 was consistently above 0.5, confirming our visual evaluation. The estimate for the same time points of the dynamic correlations of electrode 1 with electrodes 2 and 3, respectively, showed near zero values in the beginning that gradually rose and after the 50*^th^* time point, the values were mostly above 0.5. These results again confirmed our visual observation of the original time series. Finally, as expected, the WGA estimates of dynamic correlations between hippocampal and striatal LFPs were near zero for most of the time points. Thus, the WGA method led to meaningful results.

## 4. Quantifying Uncertainty and Testing Hypothesis

In this section, we present methods to obtain confidence intervals (CIs) for our dynamic correlation estimates. We also present a statistical testing framework to assess whether correlation is dynamic or not. We calculate confidence intervals based on bootstrap samples of the original pair of time series. To be more specific, we first obtain WGA dynamic correlation estimates for each bootstrap sample of the original pair, and 95% CI at each time point is then obtained by calculating the 2.5*^th^* and 97.5*^th^* percentiles among all bootstrap based estimates. Our bootstrap sampling method is based on adapting slightly the ‘multivariate linear process bootstrap’ (MLPB) method developed and presented in [27]. The core idea behind our adaptation is same as in ‘DCBootCB’ algorithm presented in [28]. The hypothesis testing framework is based on adapting the statistical tests proposed in [29]. Details of all the approaches are given below.

### 4.1. Confidence Intervals

A simple bootstrap based re-sampling will destroy the autocovariance structure of the original pair of time series. A specialized re-sampling method such as MLPB algorithm that preserves the covariance structure between and within each time series in the pair is necessary. MLPB algorithm, developed by Jentsch and Politis have been shown [27] to give consistent estimates for statistics of order two or higher, in particular consistent estimates for autocovariance and autocorrelation. We provide a summary sketch of the key ingredients involved in the MLPB algorithm. Let us denote the two time series under consideration by $X_{1}^{\left(i\right)},\ldots ,X_{T}^{\left(i\right)},\;i=1,2$ and $X_{vec}={\left[X_{1}^{\left(1\right)},\ldots ,X_{T}^{\left(1\right)},X_{1}^{\left(2\right)},\ldots ,X_{T}^{\left(2\right)}\right]}^{\prime }$ denote the vectorized version; this vector is centered in the first step. The main idea in the algorithm is to estimate the covariance matrix Γ of centered $X_{vec}$ properly, and use this estimate to de-correlate the centered $X_{vec}$. Standardized version of the de-correlated vector is just white noise and hence the standard bootstrap scheme could be used to resample from the standardized, decorrelated series. Inverse (transform) of the de-correlation operation is done to correlate the bootstrap sample pair. Thus at the end of this step we get a bootstrap sample pair that preserves the covariance structure of the original pair. The final step involves de-centering so that the bootstrap pair will have the same means as the original pair.

The key step in MLPB algorithm is the proper estimation of the covariance matrix Γ of $X_{vec}$. A naive estimate for Γ could be obtained using the sample autocovariance function C(h), with (i,j)*^th^* element being C(i−j). Here

$$C\left(h\right)=n^{-1}\;\;\;\;\;\;\;\;\;\;\;\sum_{t=max(1,1-h)}^{min(n,n-h)}\;\;\;\;\;\;\;\;\;\;C_{t+h}C_{t}^{\prime },\;\mathrm{where}\;C_{k}={\left[\left(X_{k}^{\left(1\right)}-{\bar{X}}^{\left(1\right)}\right),\left(X_{k}^{\left(2\right)}-{\bar{X}}^{\left(2\right)}\right)\right]}^{\prime },\;{\bar{X}}^{\left(i\right)}=\sum_{k=1}^{T}X_{k}^{\left(i\right)},\;i=1,2.$$

However, Jentsch and Politis [27] showed that the naive estimator is not consistent. A consistent estimator proposed in [27] is ${\hat{\Gamma }}^{\mathrm{JP}}={\left[\Gamma_{ij}^{\mathrm{JP}}\right]}_{T\times T}$, where the (i,j)*^th^* element is $\Gamma_{ij}^{\mathrm{JP}}=\kappa \left(i-j\right)C\left(i-j\right),\;i,j=1,\ldots ,T.$ Here κ is a tapering function that leaves the diagonal elements unchanged and downweights the elements farther away from the diagonal. The simplest example (the one used in the simulation study in [27], and also the one used in this paper) is the trapezoid function which leaves the diagonal, super- and supra-diagonal elements unchanged and sets all other elements to zero. This estimation procedure involves a few more technical (but easy to understand) details, mainly to ensure that ${\hat{\Gamma }}^{\mathrm{JP}}$ is positive definite. We skip these details; a reader interested in these details are referred to [28] or [27]. The steps in MLPB algorithm may be summarized as follows. 

*MLPB algorithm*:

Step 1: Let $Y_{vec}$ denote the centered $X_{vec}$ where the centering for elements in ${\left[X_{1}^{\left(1\right)},\ldots ,X_{T}^{\left(1\right)}\right]}^{\prime }$ and in ${\left[X_{2}^{\left(1\right)},\ldots ,X_{T}^{\left(2\right)}\right]}^{\prime }$ are done separately.
Step 2: Compute $W_{vec}=L^{-1}Y_{vec}$ where *L* denotes the lower left matrix in Cholesky decomposition ($LL^{\prime }$) of ${\hat{\Gamma }}^{\mathrm{JP}}$.
Step 3: Let $Z_{vec}$ be the standardized version of $W_{vec}$ obtained by subtracting the mean and dividing by the standard deviation (SD). Here mean and SD are computed from all elements in $W_{vec}$.
Step 4: Generate $Z_{vec}^{boot}$ by performing i.i.d resampling from $Z_{vec}$.
Step 5: Compute $Y_{vec}^{boot}=LZ_{vec}^{boot}$. Split $Y_{vec}^{boot}$ into two separate vectors $Y^{(i),boot},\;i=1,2$ with $Y^{(1),boot}$ containing the first *T* elements and $Y^{(2),boot}$ containing the last *T* elements of $Y_{vec}^{boot}$.
Step 6: Add mean of $X_{1}^{\left(1\right)},\ldots ,X_{T}^{\left(1\right)}$ to each element of $Y^{(1),boot}$ to obtain $X^{(1),boot}$. Similarly, add mean of $X_{1}^{\left(2\right)},\ldots ,X_{T}^{\left(2\right)}$ to each element of $Y^{(2),boot}$ to obtain $X^{(2),boot}$. The pair of vectors $X^{(1),boot}$ and $X^{(2),boot}$ are the bootstrap sample pair outputted.

Let *X* denote the 2×T data matrix with the elements of the ith row being $X_{1}^{\left(i\right)},\ldots ,X_{T}^{\left(i\right)},i=1,2.$ In order to calculate the bootstrap based confidence intervals we employ the same strategy as in ‘DCBootCB’ algorithm presented in [28]. Steps are given below. 

*BootCI algorithm*:

Step 1: Divide the data matrix X into K adjacent blocks, where each block is a 2×(T/K) matrix.
Step 2: For each block run the MLPB algorithm to get a bootstrap sample pair corresponding to that block.
Step 3: Combine the K adjacent blocks of bootstrap sample pairs and apply the WGA algorithm on the combined bootstrap sample pair.
Step 4: Repeat the above steps B times to get B dynamic correlation time series based on the WGA algorithm.
Step 5: At each time point calculate the 2.5*^th^* and 97.5*^th^* percentiles among the bootstrap samples to obtain the bootstrap based 95% confidence interval.

A computationally simpler approach to calculate the 95% confidence interval is based on Fisher’s z-transformation of the correlation coefficient:

$$z_{t}=0.5\log\left(\frac{1+\rho_{t}}{1-\rho_{t}}\right),\;t=1,\ldots ,T,$$

where $\rho_{t}$ is the estimate of dynamic correlation based on WGA at time *t*. Asymptotic standard error for $z_{t}$ is $SE\left(z_{t}\right)=1/\sqrt{T-3}$. The back transformation to obtain $\rho_{t}$ is $\rho_{t}=\tanh\left(z_{t}\right)$, where tanh represents the hyperbolic tangent function. Based on this, the corresponding 95% confidence intervals can be calculated as

$$\left(\tanh\left(z_{t}-\frac{1.96}{\sqrt{T-3}}\right),\tanh\left(z_{t}+\frac{1.96}{\sqrt{T-3}}\right)\right).$$

**Figure 12:**
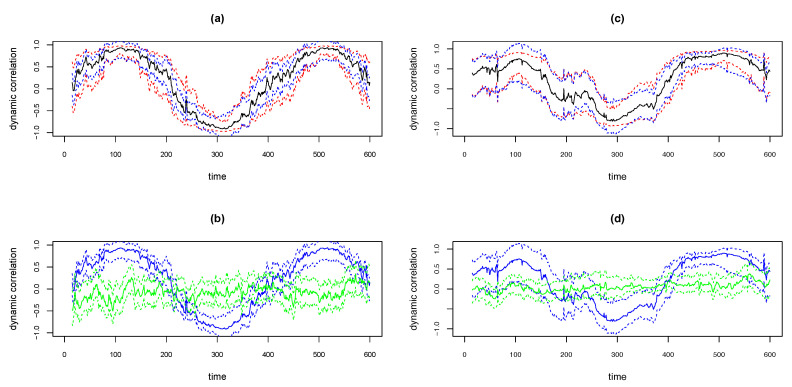
Plots of WGA estimates and 95% confidence intervals for simulated data with D2b design, with bivariate normal (**a**,**b**) and bivariate Cauchy (**c**,**d**). Upper panels plot WGA estimate (black), 95% CI with ‘BootCI’ algorithm (blue) and with z-transformation (red). Lower panels plot WGA estimate and BootCI 95% CI (both blue), and corresponding estimates for randomly permuted series (green).

We illustrate the above methods using the simulated examples shown in the top panels in Figure 12. The simulated data is based on the D2b. That is, the covariance matrix of the bivariate time series is of the form as in Equation (3), with p(t)=sin(t/Δ), Δ=1024/16=64. The sample size T=600. Thus the underlying true dynamic correlation is the same as depicted by the solid black lines in the right panels in Figure 3. The time series pair for the simulation in the left panel in Figure 12 was based on bivariate Normal and for the right panel based on bivariate Cauchy. The solid black curves in the top panels in Figure 12 are the estimates of dynamic correlation obtained using the WGA algorithm. The blue curves are the 95% confidence intervals obtained using the ‘BootCI’ algorithm and the red curves are the ones using the z-transformation method. As seen in the from the plots in the two top panels, the red and blue curves are close to each other. However, based on extensive simulations, Kudela et al. [28] determined that the coverage for the bootstrap based confidence interval is better than that based on z-transformation. We assume that the same result hold in our case too.

At any time point we may determine whether the dynamic correlation is significantly different from zero based on the bootstrap based confidence intervals and permutation based approaches described as follows. If we randomly permute both time series, the correlation between the resulting pair will be zero. We may obtain bootstrap based confidence interval for the dynamic correlation (which is an estimate of zero) between the randomly permuted pair using the ‘BootCI’ algorithm. The solid green lines in the bottom panels in Figure 12 show the WGA based dynamic correlation estimate of the randomly permuted series, and dotted green lines show the 95% confidence intervals for the corresponding solid green line using the ‘BootCI’ algorithm. The blue lines depicted in the bottom panels are same as the dynamic correlation estimate (solid line) for the original pair and the corresponding bootstrap based 95% confidence intervals (dotted lines) shown in the top panels. That is, the blue solid curve in the bottom panel is the exact replica of the black solid curve in the top panel and the blue dotted lines in the bottom panel is the exact replica of the blue dotted lines above. The time points at which the two confidence intervals do not overlap are the points at which the dynamic correlation is statistically different from zero at a 5% significance level.

### 4.2. Hypothesis Testing

In the above we presented methods to quantify uncertainty and a method to assess the time points at which the dynamic correlation is significantly different from zero. In this subsection we present a statistical hypothesis testing framework to assess whether ‘dynamic’ correlation detected is really dynamic or static. We consider the two statistical tests presented in [29] and show how they can be applied for the methods presented in this paper. The null and the alternate hypotheses under consideration are

$$H_{o}:\eta =0,\;\mathrm{versus}\;H_{a}:\eta >0,$$

where $\eta^{2}$ is the variance of the dynamic correlation time series. Thus, the null hypothesis correspond to ‘static’ case and the alternate correspond to the ‘dynamic’ case.

One obvious estimate of $\eta^{2}$ is the sample variance of estimated ${\hat{\rho }}_{t}^{\prime }s$, which is the basis for the first test statistic proposed in [29]:

$$\kappa^{2}=\frac{1}{T-ws-1}\sum_{t=ws+1}^{T}{\left({\hat{\rho }}_{t}-\hat{\bar{\rho }}\right)}^{2},\;\mathrm{where}\;\hat{\bar{\rho }}=\frac{1}{T-ws-1}\sum_{t=ws+1}^{T}{\hat{\rho }}_{t}.$$

We propose a slightly modified version of the above statistic:

$$\kappa_{\mathrm{med}}^{2}=\frac{1}{T-ws-1}\sum_{t=ws+1}^{T}{\left({\hat{\rho }}_{t}-{\hat{\rho }}_{\mathrm{med}}\right)}^{2},\;\mathrm{where}\;{\hat{\rho }}_{\mathrm{med}}=\mathrm{median}\left\{{\hat{\rho }}_{t}|t=ws+1,\ldots ,T\right\}.$$

Here ${\hat{\rho }}_{t}$ denotes the dynamic correlation estimated by the WGA method at time *t*, and ws denotes the window-size used. We also consider another statistic originally proposed by Zalesky et al. [30] which is described as follows. Let $\tau_{1},\ldots ,\tau_{J}$ denote the time points at which the series $\left\{{\hat{\rho }}_{t},t=ws+1,\ldots ,T\right\}$ crosses its median value ${\hat{\rho }}_{\mathrm{med}}$. Let $\tau_{0}=ws+1$ and $\tau_{J+1}=T$. Thus we get a partition of the interval [ws+1,T] with J+1 segments [$\tau_{j-1}$, $\tau_{j}$], j=1,…J+1, and within each segment the corresponding ${\hat{\rho }}_{t}$ is completely above the median or completely below the median. Let $I_{j}=\tau_{j}-\tau_{j-1}$ denote the length of the jth interval and define

$$H_{j}=\max\{\;|{\hat{\rho }}_{t}-{\hat{\rho }}_{\mathrm{med}}\left|:t\in \left[\tau_{j-1},\tau_{j}\right]\right\}.$$

Then the test statistic proposed by Zalesky et al. [30] is

$$S=\sum_{j=1}^{J+1}I_{j}^{\alpha }H_{j}^{\beta },$$

where α and β are tuning parameters. Zalesky et al. [30] recommended setting α=0.9 and β=1.

Hypothesis testing can be done based on the standard error (SE) of each test statistic calculated from bootstrap samples obtained from the original pair of time series. Lower limit of 95% confidence intervals can be obtained by subtracting 2.5*^th^* percentile value and adding 97.5*^th^* percentile to the corresponding test statistic; here, the percentiles are obtained from among the test statistic values calculated from bootstrap samples. Null hypothesis may be rejected if the 95% confidence interval does not contain zero.

We illustrate the strategy for the simulated data in Figure 12 right panels (i.e., design D2b and bivariate Cauchy; dynamic correlation estimated by WGA). The 95% confidence interval (CI) calculated for the $\kappa_{\mathrm{med}}^{2}$ test statistic for the above simulated example was (0.221, 0.679) based on which we may reject the null hypothesis since the CI does not contain zero. For the second test statistic, optimal values of α and β may be obtained also from bootstrap samples. For α and β, we considered a grid ranging from 0.05 to 1.50 with step size 0.05, and for each (α, β) pair in the 2-dimensional grid calculated the sum-squared error based on the bootstrap samples. We picked the optimal (α, β) pair as the one for which the bootstrap based sum-squared error was minimal. For the simulated example above, we obtained the optimal α=0.05 and β=1.5. 95% confidence interval for the second test statistic based on this (α, β) pair was (0.982, 6.659). Since this interval does not contain zero, we reject the null hypothesis based on the second test statistic as well. Thus, for the simulation example, we correctly reject the null hypothesis of ‘no dynamic correlation’ based on both hypothesis testing strategies presented in this section.

## 5. Conclusions and Discussion

Understanding the dynamic correlation underlying a pair of time series is of interest in many areas within neuroscience. Currently, existing techniques such as sliding window (SW) or GARCH-based DCC can be applied to this estimation problem. Linquist and co-authors [10] evaluated the existing methods and concluded that DCC is an attractive option for effective dynamic correlation estimation in fMRI data. Our analysis confirmed that DCC performs well for nicely behaved time series from a bivariate normal distribution. However, for time series with extreme values, common in neuroscience, DCC’s performance was sub-optimal. Our analyses also revealed that in practice DCC estimation is prone to numerical instability. To address these problems, in this paper, we proposed a novel approach to the estimation of dynamic correlation based on a weighted graph.

To study how our new method, the WGA, compares with the SW and DCC methods, we conducted extensive simulations. Results from our simulation study showed that WGA’s performance was superior to the existing methods in the presence of extreme values, especially when underlying correlation is truly dynamic. We also compared the results obtained by applying the three methods to one real fMRI data set and to one LFP data set with known properties and extreme values recorded in an awake rat. These analyses indicated that the WGA based method led to more biologically meaningful results and performed better in the presence of outliers than SW or DCC. We also provided a theoretical framework to better understand our method and presented approaches to quantify uncertainty and test hypotheses.

One factor that may contribute to the optimal performance of the WGA method may be the combination of local and global aspects of its algorithm. Method #1 (SW) is based on only local information - correlation at each time point is based only on the values within the sliding window. As mentioned in Lindquist et al. [10], in these conditions the dynamic profiles of even random noise may show compelling changes in correlation across time. The local nature of SW could be transformed into a more global nature by increasing the window-size, but increasing the window-size beyond a particular level will lead to the method overlooking real dynamic changes. Method #2 is much more of a global estimation approach in the sense that the entire data is used for estimating the parameters in DCC. The new approach based on WGA incorporates both local and global components. Since it is window-based, it is partly local in nature like SW. However, for time points within each window the correlations calculated involve median weight vectors with similar length as that of the original time series and consisting of weights from all time points from the entire series. This approach confers global character to the new method. The combined local and global aspects of the WGA method may result in optimal performance on data sets that do not conform to normality and contain extreme values. Our simulation study shows that extreme values do not pose a problem for WGA, which is based on a weighted graph where the weights are calculated using an *arctan* function. *Arctan*, like other functions used in robust estimation such as Huber’s function, trimming function or Tukey’s bisquare function reduces the influence of outlying values by applying a cap (see section 14.5 in [31]). Variations of our method could involve these other functions from the robust statistics literature.

Recently, a copula based dynamic conditional correlation approach for estimating time-varying correlation was proposed in [32]. Copulas are multivariate cumulative distribution functions that could be used to capture distribution-free dependence structure among random variables [33]. As pointed out in [32], copula framework can be combined with DCC-GARCH model to alleviate the sensitivity of DCC-GARCH approach on distributional assumptions. Based on a simulation study, it was showed in [32] that when bivariate time series data are generated from lognormal or beta densities, the copula-DCC-GARCH approach shows substantial improvement over DCC-GARCH method alone. In addition to the difference in the types of densities considered in [32], the design for simulations was also quite different: underlying dynamic correlation considered in their paper was much more rapidly varying than those considered in our paper or in [10]. In this regard, we acknowledge that the simulation study is limited in this introductory paper in which we present our new approach. Second, for all simulations in our paper, we considered only one fixed window size (ws = 15). Extensive empirical studies comparing the outcomes of SW, DCC, copula-DCC, and VGA methods while varying the density functions, the rate of change of the underlying dynamic correlation, and window size will be needed in order to provide comprehensive recommendations for the practitioner aiming to utilize one of these analyses.

Building upon the mapping of multivariate time series into a multilayer graph proposed in [34], Sannino and co-authors described an approach to capture modulations across resting-state networks [35]. Sannino et al. observed that the networks within the multiplex layers have a modular structure which is an indication of different temporal regimes. Partitioning the visibility graph based on these modules provides a natural decomposition of the time series into time intervals. The approach presented in [35] is to capture the similarity between neural events using Sörensen similarity index between each pair of modules in the two time series. One difference from our approach is that we did not use a visibility criterion; another difference is that the approach in [35] is based on decomposing time series into time intervals. As in our case, we consider the approach in [35] could also be robust towards extreme values. Systematically exploring the differences and similarities between these two approaches will be an interesting topic for future work.

In this study, we used a task-based fMRI dataset to validate our method. Since the dataset is task-based, the dynamics may have been emphasized when compared to fMRI acquisition without tasks, namely resting state fMRI. It is a limitation of our work that we did not validate our finding by incorporating resting state fMRI data, but the Stroop task on which our dataset was based, is irrelevant for the method we proposed. In other words, we did not use the method to explore functional modifications introduced by performing the Stroop task, but we used fMRI data generated during the Stroop task to show that our method can capture such functional modifications. It is anticipated that similar validation can be obtained from resting state data.

Focusing on the pairwise bivariate correlations alone may exclude the potential effects of other regions on the pair of brain regions under consideration. There are new directions proposed recently in the neuroscience literature using partial correlations to account for such confounding effects [36,37]. To control for false positives due to partial correlations in the presence of colliders, [36,37] proposed a combined approach that includes both partial correlations and pairwise bivariate correlations. The approach presented in this paper focused only on bivariate correlations. Extending our method to a combined approach that takes into account confounding effects will also be an interesting direction for future work.

## Figures and Tables

**Table 1:** Bivariate Normal in design D1.

	Mean of Absolute Value of Correlations Across Time			
	**T = 150**	**T = 300**	**T = 600**	**T = 1000**
SW	0.219(0.038)	0.218(0.027)	0.218(0.019)	0.218(0.014)
WGA	0.134(0.033)	0.129(0.022)	0.127(0.015)	0.126(0.012)
DCC	0.083(0.052)	0.059(0.034)	0.042(0.025)	0.033(0.019)
	**Maximum of Absolute Value of Correlations Across Time**			
	**T = 150**	**T = 300**	**T = 600**	**T = 1000**
SW	0.615(0.090)	0.669(0.076)	0.716(0.062)	0.741(0.055)
WGA	0.394(0.076)	0.424(0.066)	0.456(0.059)	0.477(0.052)
DCC	0.199(0.164)	0.164(0.138)	0.131(0.111)	0.105(0.092)

**Table 2:** Bivariate Cauchy in design D1.

	Mean of Absolute Value of Correlations Across Time			
	**T = 150**	**T = 300**	**T = 600**	**T = 1000**
SW	0.526(0.079)	0.529(0.057)	0.530(0.040)	0.529(0.031)
WGA	0.241(0.097)	0.220(0.070)	0.209(0.047)	0.203(0.036)
DCC	0.338(0.155)	0.252(0.129)	0.192(0.099)	0.148(0.081)
	**Maximum of Absolute Value of Correlations Across Time**			
	**T = 150**	**T = 300**	**T = 600**	**T = 1000**
SW	0.972(0.030)	0.987(0.010)	0.992(0.005)	0.994(0.003)
WGA	0.535(0.117)	0.552(0.100)	0.578(0.086)	0.593(0.073)
DCC	0.801(0.267)	0.728(0.312)	0.657(0.323)	0.588(0.329)

**Table 3:** Results from Stroop data analysis.

Mean Value of Correlations Across Time			
	**SW**	**WGA**	**DCC**
Amygdala vs. Caudate Head	0.075	0.031	1.000
Amygdala vs. Frontal Medial Cortex	0.210	0.173	1.000
Amygdala vs. Heschel’s Gyrus	0.138	0.117	1.000
Amygdala vs. Insula	0.022	−0.020	1.000
Amygdala vs. Subcallosal Cortex	0.417	0.378	1.000
Caudate Head vs. Frontal Medial Cortex	0.280	0.301	1.000
Caudate Head vs. Heschel’s Gyrus	0.010	0.039	1.000
Caudate Head vs. Insula	0.214	0.223	1.000
Caudate Head vs Subcallosal Cortex	0.174	0.155	1.000
Frontal Medial Cortex vs. Heschel’s Gyrus	0.219	0.275	1.000
Frontal Medial Cortex vs. Insula	0.151	0.179	1.000
Frontal Medial Cortex vs. Subcallosal Cortex	0.440	0.501	1.000
Heschel’s Gyrus vs. Insula	0.619	0.622	1.000
Heschel’s Gyrus vs. Subcallosal Cortex	0.274	0.313	1.000
Insula versus Subcallosal Cortex	0.142	0.142	1.000

**Figure A6:**
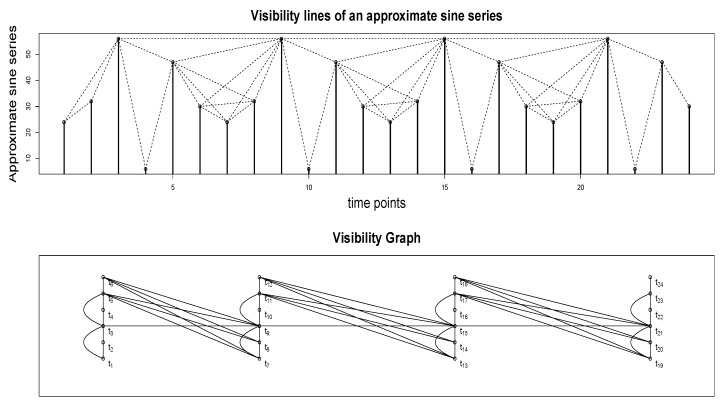
A time series and the corresponding visibility graph. $t_{1},t_{2}$ and so forth denote the time points as well as the corresponding nodes in the visibility graph.

## Appendix A. Figures and Tables

**Table A1:** Results from Stroop Data Analysis.

Maximum Value of Correlations Across Time			
	**SW**	**WGA**	**DCC**
Amygdala vs. Caudate Head	0.823	0.507	1.000
Amygdala vs. Frontal Medial Cortex	0.882	0.666	1.000
Amygdala vs. Heschel’s Gyrus	0.657	0.504	1.000
Amygdala vs. Insula	0.767	0.584	1.000
Amygdala vs. Subcallosal Cortex	0.830	0.692	1.000
Caudate Head vs. Frontal Medial Cortex	0.795	0.701	1.000
Caudate Head vs. Heschel’s Gyrus	0.606	0.492	1.000
Caudate Head vs. Insula	0.736	0.602	1.000
Caudate Head vs Subcallosal Cortex	0.800	0.624	1.000
Frontal Medial Cortex vs. Heschel’s Gyrus	0.757	0.629	1.000
Frontal Medial Cortex vs. Insula	0.789	0.684	1.000
Frontal Medial Cortex vs. Subcallosal Cortex	0.886	0.831	1.000
Heschel’s Gyrus vs. Insula	0.892	0.851	1.000
Heschel’s Gyrus vs. Subcallosal Cortex	0.817	0.703	1.000
Insula versus Subcallosal Cortex	0.693	0.599	1.000

## Appendix B. Visibility Criteria

Visibility graph algorithm (VGA) is a method that extends usefulness of the techniques and focus of mathematical graph theory to characterize time series. It has been shown that the visibility graph inherits several properties of the time series [16,38], and its study reveals nontrivial information about the time series itself.

Figure A6 top panel illustrates how the visibility algorithm works. The time series plotted in the upper panel is an approximate sine series; specifically, a sine series with Gaussian white noise added. The values at 24 time points are plotted as vertical bars. One may imagine these vertical bars as, for example, buildings along a straight line in a city landscape (i.e., a city block). Each node in the associated visibility graph (shown in the bottom panel) corresponds to each time point in the series. So, the graph in Figure A6 has 24 nodes. We draw a link or an edge between a pair of nodes, say $t_{i}$ and $t_{j}$, if the visual line of sight from the top of the building (vertical bar) situated at $t_{i}$ towards the top of the building/bar at $t_{j}$ is not blocked by any intermediate buildings - that is, if we were to draw a line from the top of the vertical bar at $t_{i}$ to the top of the vertical bar at $t_{j}$, it should not intersect any intermediate vertical bars. Visibility lines corresponding to the edges in the graph are plotted as dotted lines in the figure in the upper panel. For example, there is no edge between $t_{2}$ and $t_{4}$ since the line of sight (not shown) between the top points of the vertical bars at these two time points is blocked by the vertical bar at $t_{3}$. On the other hand, there is an edge between $t_{1}$ and $t_{3}$ since the corresponding visibility line (shown as a dotted line) does not intersect the vertical bar at $t_{2}$.

More formally, the following visibility criteria can be established: two arbitrary data values ($t_{q}$, $y_{q}$) and ($t_{s},y_{s}$) will have visibility, and consequently will become two connected nodes of the associated graph, if any other data ($t_{r},y_{r}$) placed between them fulfills:

$$y_{r}<y_{s}+\left(y_{q}-y_{s}\right)\frac{t_{s}-t_{r}}{t_{s}-t_{q}}.$$

In our new approach both time series were converted into graphs using a modified version of the weighted visibility graph algorithm (WVGA), introduced in Supriya et al. [15]. WVGA is an extension of the visibility graph algorithm (originally developed by Lacasa et al. [16]) that converts a time series to a graph. The nodes of the WVGA-based graphs correspond to the time points and therefore, the graph generated from each time series consists of *T* nodes. The weight $w_{ab}$ of the edge between the nodes corresponding to time points $t_{a}$ and $t_{b}$, with a<b, is calculated using Equation (2) given in p.6558 in Supriya et al. [15]:

$$w_{ab}=\arctan\frac{x\left(t_{b}\right)-x\left(t_{a}\right)}{t_{b}-t_{a}}.$$

Here $x(t_{a})$ and $x(t_{b})$ are the values of the time series at time points $t_{a}$ and $t_{b}$, respectively. All edge weights are calculated in radians.

We modified WVGA in several ways. First, strictly speaking, for the graph to be a visibility graph, there should be no edge between two nodes if there is no “visibility" between them, where “visibility" is defined based on the criterion spelled out above. However, in our version of the algorithm we kept all the edges, and each edge had an assigned weight based on the arctan function mentioned above. Second, while Supriya et al. [15] used only the absolute values of the weights, we retained the signs of the weights calculated, as is. The reason we introduced these modifications is that the shape of the arctan function, rather than the visibility criterion, provides a suitable solution for our purpose.

## Appendix C. Theoretical Considerations

### Appendix C.1. Heuristic Justification

A heuristic justification based on an approximation argument for the new approach (WGA) is as follows. Let $x\equiv \{x_{t}\}$ and $y\equiv \{y_{t}\}$ denote the first and second time series. Arctan function and median filtering in WGA were used to robustify the method. If we ignore the arctan function in the algorithm for a moment, and use mean instead of median, then the $t^{th}$ element in $W_{\left(i:\;\mathrm{median}\right)}^{\left(1\right)}$ (–in this case it should be called $W_{\left(i:\;\mathrm{mean}\right)}^{\left(1\right)}$, rather–) is

$$\begin{array}{c} \mathrm{mean}\left\{\frac{x_{t}-x_{i}}{t-i},\frac{x_{t-1}-x_{i}}{t-1-i},\ldots ,\frac{x_{t-\Delta +1}-x_{i}}{t-\Delta +1-i}\right\}\\ \;\;\;=\Delta^{-1}\left\{\frac{x_{t}-x_{i}}{t-i}+\frac{x_{t-1}-x_{i}}{t-1-i}+\ldots +\frac{x_{t-\Delta +1}-x_{i}}{t-\Delta +1-i}\right\}=u_{it}\left(\mathrm{say}\right). \end{array}$$

Here Δ is the window size (ws) that we used in the WGA algorithm. Note that we also assumed spacing $t_{j}-t_{i}=j-i$ for convenience. Re-arranging and collecting terms, we may write

$$u_{it}=X^{t,\Delta }-x_{i}C_{t,i},\;\mathrm{where}\;X^{t,\Delta }=\Delta^{-1}\sum_{k=0}^{\Delta -1}{\left(t-k-i\right)}^{-1}x_{t-k}\;\mathrm{and}\;C_{t,i}=\Delta^{-1}\sum_{k=0}^{\Delta -1}{\left(t-k-i\right)}^{-1}.$$

Similarly, the $t^{th}$ element in $W_{\left(i:\;\mathrm{median}\right)}^{\left(2\right)}$ (–$W_{\left(i:\;\mathrm{mean}\right)}^{\left(2\right)}$, rather-) may be written as

$$v_{it}=Y^{t,\Delta }-y_{i}C_{t,i},\;\mathrm{with}\;Y^{t,\Delta }=\Delta^{-1}\sum_{k=0}^{\Delta -1}{\left(t-k-i\right)}^{-1}y_{t-k}.$$

We also write

$${\bar{u}}_{t}=T^{-1}\sum_{i=1}^{T}u_{it}={\bar{X}}^{t,\Delta }-m_{t}^{x}\;\mathrm{and}\;{\bar{v}}_{t}=T^{-1}\sum_{i=1}^{T}v_{it}={\bar{Y}}^{t,\Delta }-m_{t}^{y},$$

$$\mathrm{where}\;m_{t}^{x}=T^{-1}\sum_{i=1}^{T}x_{i}C_{t,i}\;\mathrm{and}\;m_{t}^{y}=T^{-1}\sum_{i=1}^{T}y_{i}C_{t,i}.$$

Thus, without the arctan function, and with mean instead of median in the algorithm, our estimate for dynamic correlation at time *t* will be

(A1) $${\hat{\rho }}_{t}=\frac{\sum_{i=1}^{T}\left(u_{it}-{\bar{u}}_{t}\right)\left(v_{it}-{\bar{v}}_{t}\right)}{\sqrt{\sum_{i=1}^{T}{\left(u_{it}-{\bar{u}}_{t}\right)}^{2}\sum_{i=1}^{T}{\left(v_{it}-{\bar{v}}_{t}\right)}^{2}}}.$$

The $C_{t,i}$ term depends on *i* and hence cannot be factored out of the sums in the numerator and denominator. For a heuristic argument, if we take $C_{t,i}$ to be approximately a constant that depends only on *t*, say $C_{t}$, and also assume $X^{t,\Delta }\approx {\bar{X}}^{t,\Delta }$ and $Y^{t,\Delta }\approx {\bar{Y}}^{t,\Delta }$, then $C_{t}^{2}$ term in the numerator and the denominator of Equation (A1) cancels out, so that in the case without arctan and with mean instead of median we get

$${\hat{\rho }}_{t}\approx \mathrm{Corr}\left(x,y\right).$$

This ${\hat{\rho }}_{t}$ does not depend on *t* (i.e., not dynamic) but it is in the ballpark because it is an estimate of the overall average correlation across all time points. If we use median instead of mean, the $C_{t,i}^{2}$ term does not cancel out in Equation (A1) and hence the final estimate will depend on *t* (i.e., will be dynamic) and the arctan function will make the method more robust to outliers. We emphasize that the arguments presented in this subsection is just heuristic and the real justification of our algorithm is based on empirical simulations that we conducted and presented in the paper.

### Appendix C.2. A Theoretical Framework

We present a theoretical framework to better understand our estimate for dynamic correlation. For convenience with notation, let us write $t_{i}=i$. In the new notation, the two time series under consideration are $x=\{x_{1},\ldots ,x_{T}\}$ and $y=\{y_{1},\ldots ,y_{T}\}$. We also assume for convenience that the two time series are centered. Consider the function $\Phi^{x}:x\subset R\to R^{T}$ defined by

$$\Phi^{x}\left(x_{i}\right)={\left[\frac{x_{1}-x_{i}}{1-i},\frac{x_{2}-x_{i}}{2-i},\ldots ,0,\ldots ,\frac{x_{T}-x_{i}}{T-i}\right]}^{\prime }$$

with 0 at the ith spot, and the function $k^{xx}:x\times x\to R$ defined by

$$k^{xx}\left(x_{i},x_{j}\right)=\langle \Phi^{x}\left(x_{i}\right),\Phi^{x}\left(x_{j}\right)\rangle =\sum_{t=1,t\neq i,j}^{T}\frac{\left(x_{t}-x_{i}\right)\left(x_{t}-x_{j}\right)}{\left(t-i)(t-j\right)}.$$

Similarly define $\Phi^{y}:y\subset R\to R^{T}$ as

$$\Phi^{y}\left(y_{i}\right)={\left[\frac{y_{1}-y_{i}}{1-i},\frac{y_{2}-y_{i}}{2-i},\ldots ,0,\ldots ,\frac{y_{T}-y_{i}}{T-i}\right]}^{\prime },$$

$$k^{yy}\left(y_{i},y_{j}\right)\equiv \langle \Phi^{y}\left(y_{i}\right),\Phi^{y}\left(y_{j}\right)\rangle =\sum_{t=1,t\neq i,j}^{T}\frac{\left(y_{t}-y_{i}\right)\left(y_{t}-y_{j}\right)}{\left(t-i)(t-j\right)},$$

and

$$k^{xy}\left(x_{i},y_{j}\right)\equiv \langle \Phi^{x}\left(x_{i}\right),\Phi^{y}\left(y_{j}\right)\rangle =\sum_{t=1,t\neq i,j}^{T}\frac{\left(x_{t}-x_{i}\right)\left(y_{t}-y_{j}\right)}{\left(t-i)(t-j\right)}=\langle \Phi^{y}\left(y_{j}\right),\Phi^{x}\left(x_{i}\right)\rangle \equiv k^{yx}\left(y_{j},x_{i}\right).$$

Let

$$K={\left[\begin{array}{cc} K^{xx} & K^{xy}\\ K^{yx} & K^{yy} \end{array}\right]}_{2T\times 2T}$$

where $K^{xx}$, $K^{xy}$, $K^{yx}$ and $K^{yy}$ are T×T matrices defined as

$${\left[K^{xx}\right]}_{ij}=k^{xx}\left(x_{i},x_{j}\right),\;\;{\left[K^{yy}\right]}_{ij}=k^{yy}\left(y_{i},y_{j}\right),\;\;{\left[K^{xy}\right]}_{ij}={\left[K^{yx}\right]}_{ji}=k^{xy}\left(x_{i},y_{j}\right).$$

Based on the above definitions, we have the following lemma.

**Lemma** **A1.**
*K is symmetric and positive semidefinite.*

**Proof.** Symmetry is easy to check using the definition. To check positive semi-definiteness, let $\nu ={\left[\alpha^{\prime },\beta^{\prime }\right]}^{\prime }$ be a 2T-vector of real numbers, where $\alpha ={\left[\alpha_{1},\ldots ,\alpha_{T}\right]}^{\prime }$ and $\beta ={\left[\beta_{1},\ldots ,\beta_{T}\right]}^{\prime }$. Then

$$\nu^{\prime }K\nu =∥\sum_{i=1}^{T}\left(\alpha_{i}\Phi^{x}\left(x_{i}\right)+\beta_{i}\Phi^{y}\left(y_{i}\right)\right){∥}^{2}\geq 0.$$

 □

Note that our dynamic correlation estimate ${\hat{\rho }}_{t}$ sans arctan and median filtering is ${\hat{\rho }}_{t}={\left[K^{xy}\right]}_{tt}/\sqrt{{\left[K^{xx}\right]}_{tt}{\left[K^{yy}\right]}_{tt}}.$ Framing our estimate in the above manner makes it easy to check that it is a proper correlation estimate that takes values in [−1,1].

**Lemma** **A2.**
*$-1\leq {\hat{\rho }}_{t}\leq 1$, for all t=1,…T.*

**Proof.** Proof follows easily from the fact that for any symmetric positive semidefinite matrix *M*, $M_{ij}^{2}\leq M_{ii}M_{jj}$, since all principal submatrices of a positive semidefinite matrix are also positive semi-definite (see p.398 in [39]). Here $M_{ij}$ denotes the $ij^{th}$ element of *M*. (Proof also follows from a simple application of Cauchy-Schwartz inequality directly to $\Phi^{x}\left(x_{i}\right)$ and $\Phi^{y}\left(y_{i}\right)$, which does not require the matrix framework.) □

Note that $\Phi^{x}$, $\Phi^{y}$ are “projections” into a larger dimensional feature space. Although the theoretical framework that we outlined above has some similarities with kernel method framework in machine learning, the two frameworks are not the same. The $\Phi^{x}$ and $\Phi^{y}$ functions in our framework are *known* functions, while as in the kernel setting, they are typically unknown functions. Also, $k^{xx}$, $k^{yy}$ and $k^{xy}$ are not exactly kernel functions because they do not depend on their inputs alone. For instance, in order to evaluate $k^{xx}\left(x_{i},x_{j}\right)$ we use all other $x_{t}$ (t≠i,j) as well. Hence, in particular, we cannot use the “kernel trick”. Nevertheless *K* is still symmetric and positive semidefinite like a Gram matrix and it is interesting to observe that our dynamic correlation estimate is a true correlation estimate in a higher dimensional space.
